# Single-sample image-fusion upsampling of fluorescence lifetime images

**DOI:** 10.1126/sciadv.adn0139

**Published:** 2024-05-23

**Authors:** Valentin Kapitany, Areeba Fatima, Vytautas Zickus, Jamie Whitelaw, Ewan McGhee, Robert Insall, Laura Machesky, Daniele Faccio

**Affiliations:** ^1^ School of Physics & Astronomy, University of Glasgow, Glasgow G12 8QQ, UK.; ^2^ Department of Laser Technologies, Center for Physical Sciences and Technology, LT-10257 Vilnius, Lithuania.; ^3^ Cancer Research UK, Beatson Institute, Glasgow, UK.

## Abstract

Fluorescence lifetime imaging microscopy (FLIM) provides detailed information about molecular interactions and biological processes. A major bottleneck for FLIM is image resolution at high acquisition speeds due to the engineering and signal-processing limitations of time-resolved imaging technology. Here, we present single-sample image-fusion upsampling, a data-fusion approach to computational FLIM super-resolution that combines measurements from a low-resolution time-resolved detector (that measures photon arrival time) and a high-resolution camera (that measures intensity only). To solve this otherwise ill-posed inverse retrieval problem, we introduce statistically informed priors that encode local and global correlations between the two “single-sample” measurements. This bypasses the risk of out-of-distribution hallucination as in traditional data-driven approaches and delivers enhanced images compared, for example, to standard bilinear interpolation. The general approach laid out by single-sample image-fusion upsampling can be applied to other image super-resolution problems where two different datasets are available.

## Teaser

A computational approach to microscopy enables upsampling fluorescence lifetime images by factors as high as 16 × 16.

## INTRODUCTION

Fluorescence lifetime imaging microscopy (FLIM) finds extensive applications in biological studies where the lifetimes of fluorophores can be used as indicators of the cellular metabolism (*1*–*5*), cellular environment (*6*–*8*), or changes in molecular conformation visible through Förster resonance energy transfer, enabling measurement of protein:protein interactions during processes such as cellular signalling (*9*–*12*). In medical settings, endogenous FLIM can be used for identifying cancerous tissue (*13*, *14*).

FLIM setups excite a sample with short-wavelength light and measure the temporal profile of long-wavelength fluorescence from the sample (*15*). Excitation is achieved using a pulsed or amplitude modulated laser for time-domain and frequency-domain FLIM, respectively (*11*), while emission is usually collected with time-correlated single-photon counting (TCSPC) or time-gated hardware. Fluorescence lifetime is then recovered from the temporal decay of fluorescence emission. Popular lifetime estimation schemes include least-squares deconvolution (*16*), Laguerre expansion (*17*), phasor fitting (*2*, *3*), rapid lifetime determination (*18*, *19*), center-of-mass estimation (*20*, *21*), and machine learning (*22*–*24*).

Images are formed through raster-scanning or wide-field detection. Scanning systems allow confocal or two-photon microscopy setups, giving excellent image resolution and aligning well with TCSPC methods that give rich fluorescence information. However, scanning also presents drawbacks, such as the lack of instantaneous complete field-of-view (FOV) information and long acquisition times that are incompatible with the rapid intracellular dynamics of living cells (*25*, *26*). Wide-field systems overcome these challenges by measuring temporal decay from the full FOV in parallel, often using time-gated cameras such as intensified charge-coupled devices (*27*, *28*), externally gated devices (*29*, *30*), or single-photon avalanche diode (SPAD) arrays (*23*, *31*). However, intensified charge-coupled device resolution is limited by the intensifier point spread function, while SPAD arrays typically have low-pixel counts and/or low fill factors.

Computational super-resolution (SR) provides a route to overcome the trade-off between acquisition time and spatial resolution by offloading imaging from optics onto software. SR takes an undersampled image of a scene and estimates its high-resolution features. Multiple flavors of SR exist, which are generally interpolation, reconstruction (inverse retrieval), or example (learning)–based.

Interpolation is the simplest form of upsampling, encompassing several methods for connecting data points with some curve (*32*). For images, this ranges from simple schemes such as nearest, bilinear, and bicubic interpolations, through frequency-based approaches sinc and Lanczos interpolation, to covariance-based algorithms such as kriging (Gaussian processes) (*33*). While interpolation is fast and computationally inexpensive, it does not add new information to the image.

Reconstruction-based modeling instead manipulates the detection to optically redistribute information about the high-resolution target into fewer measurements. This encoding provides a mathematical forward model that is used to reconstruct the nonsampled points in an inverse retrieval framework, for example, via point spread function engineering (*34*), blurring (*35*), or compressed sensing (*36*–*39*).

Last, example-based schemes rely on computation and pattern recognition to upsample images in a data-driven manner (*40*). Classical approaches include neighbor embedding (*41*), sparse coding (*42*), and anchored neighborhood regression (*43*). More recently, machine learning algorithms have seen widespread adoption for SR (*44*, *45*). These range from SR convolution neural networks (*46*, *47*), through generative adversarial networks (*48*, *49*), to diffusion models (*50*). However, learning-based schemes traditionally need large, diverse training datasets, which can pose a bottleneck in niche fields such as FLIM; further, different fluorophores behave differently, hampering generalisation in traditional machine learning methods (*51*). Self-similarity–based SR (*52*) and self-supervised clustering (*53*) approaches offer an alternative to external training set, deriving statistical information for SR from the very image that is upsampled.

Data from different sensing modalities can yield more information about a subject than those contained in each modality alone (*45*, *54*). Fusion-based inference is a growing field with applications from medical imaging using positron emission tomography and magnetic resonance imaging (*55*), through autonomous driving using camera and light detection and ranging (*56*), to content classification using video and text (*57*). Data fusion has been applied to FLIM, by interpolating lifetime images and weighting them with intensity images for visualization (*24*, *58*).

Here, we introduce an SR method that relies on the fusion of two images: a high-resolution intensity image (no lifetime information) and a low-resolution lifetime image. Our method is called “single-sample image-fusion upsampling” (SiSIFUS). SiSIFUS generates data-driven lifetime priors matching the resolution of the intensity image; this is relatively easy and inexpensive to acquire at high resolution compared to FLIM images.

Crucially, our method generates “single-sample” priors: All information in our scheme comes from the given FOV, not external training data. We develop two priors, which extract this information from the FLIM–intensity image pair in different ways. Local priors (LPs) correlate low-resolution FLIM pixels with corresponding intensity pixels in small neighborhoods. Global priors (GPs) instead exploit morphological signatures in the image, using a neural network to predict fluorescence lifetime from intensity patches.

SiSIFUS combines data fusion and self-supervised learning into a practical SR framework. Similar to example-based self-similarity approaches, it avoids complex hardware modifications and external training data. Similar to reconstruction-based modeling, we optically measure high-resolution features, giving more information than those available in the low- resolution images alone.

## RESULTS

### Forward and inverse models

We apply SiSIFUS to both raster-scanning and wide-field FLIMs. The scanning system uses a photomultiplier tube to gather both the FLIM and intensity image, while the wide-field system uses an SPAD array to measure FLIM and a complementary metal-oxide semiconductor (CMOS) camera to measure intensity. Both setups are detailed in Materials and Methods.

SiSIFUS involves two measurements. The first is the time-resolved, low-resolution datacube, *r* ∈ ℕ^*m*,*n*,*t*^, where *m* and *n* denote the spatial positions and *t* denotes the time. The fluorescence lifetime image, τ_LR_ ∈ ℝ^*m*,*n*^, is estimated from *r* via a standard least-squares deconvolution; other schemes, such as phasor analysis or center-of-mass estimation, could be used equivalently.

The second measurement is the high–spatial resolution intensity measurement, *I* ∈ ℕ^*M*,*N*^, where *M* and *N* denote the pixel numbers of the high–spatial resolution sensor. SiSIFUS then super-resolves the lifetime image τ_LR_ to match the pixel count of the intensity image *I*.

**Fig. 1.**
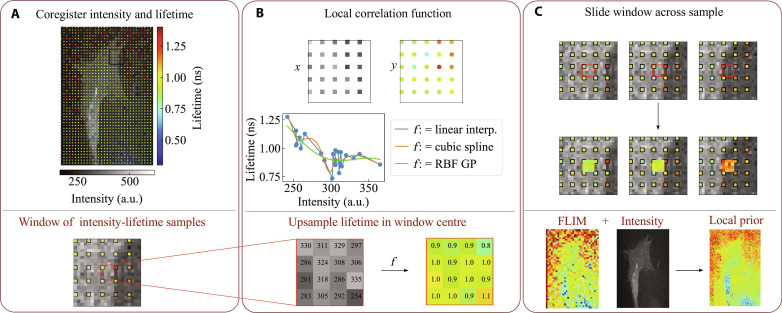
Schematic of the LP method. (**A**) Shown is a CMOS (fluoresence intensity) FOV, with the SPAD FOV (fluorescence lifetime), overlayed on top of it so as to match the sparse, low–fill factor pixel layout of the SPAD array. a.u., arbitrary units. (**B**) We zoom in on a 5 × 5 window. All SPAD pixels have a corresponding CMOS measurement, but so do the areas in-between SPAD pixels. We aim to find the lifetime at points with no SPAD samples. For this, we fit a function, for instance, linear interpolation, a cubic spline or a radial basis function Gaussian process. Then, the high-resolution CMOS pixels *x*_HR_ that we wish to upsample are fitted with this function, producing a lifetime estimate ${\hat{\tau }}_{\text{HR}}.$ (**C**) We slide the window across the FOV, fitting new functions for each new window and predicting the centers, upsampling the FLIM image to the resolution of the intensity image, window by window.

**Fig. 2.**
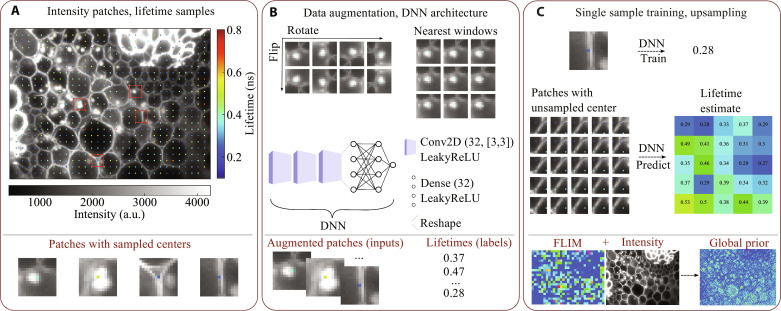
Schematic of the GP method. (**A**) Fluorescence intensity of a *Convallaria* rhizome sample stained with acridine orange, with 8 × 8 sparse lifetime samples overlayed. We extract intensity patches from this image; a few of them correspond to a central lifetime sample. These patches are training data, which we can use to predict the central lifetime of the rest of the patches. (**B**) Training inputs (patches) are augmented via rotation and mirroring. They can be further augmented by adding the patches that are nearest neighbors of training patches and allocating them the same label (lifetime) as the sampled patch. The deep neural network (DNN) architecture is simple, consisting of three two-dimensional convolutional layers, followed by three fully connected layers. (**C**) Last, the trained DNN evaluates patches with unsampled centers, thus super-resolving the lifetime image.

Our setups sample fluorescence lifetime sparsely across the FOV. In the wide-field setup, this arises from low–fill factor, ergo large dead spaces between the active areas of the SPAD pixels. In the scanning setup, this arises from the large sampling period relative to the spot size of the excitation beam in the object plane. We also assume that the intensity measurement has ~100% fill factor. Figures 1A and 2A depict how intensity and lifetime are sampled.

For 256 × 256–sized high-resolution intensity image *I* of the sample, the acquired dataset is integrated along the time axis. In a practical scenario for upsampling a confocal scan image, the FLIM samples would be acquired by taking a large line average of low-resolution scans. A large line average is needed for the fitted lifetime to have decent signal-to-noise ratio (SNR). The intensity image has decent SNR even with just a few line averages; therefore, the high-resolution intensity image could be obtained without adding an external sensor, by simply scanning a second time, with a higher pixel count but much fewer line averages.

Consequently, τ_LR_ is decimated (sparsely sampled) from the high-resolution fluorescence lifetime target, τ_HR_ ∈ *ℝ*^*M*,*N*^, that we aim to reconstruct

$$\tau_{\text{LR}}=A\tau_{\text{HR}}$$ (1)

where **A** represents the sparse sampling (decimation).

We feed the two images, *I*(*M*, *N*) and τ_LR_(*m*, *n*), to our prior-generation pipeline (explained below), which outputs LP and GP, ${\hat{\tau }}_{\text{LP}}\left(M,N\right)$ and ${\hat{\tau }}_{\text{GP}}\left(M,N\right)$ , respectively. These priors constrain an (otherwise ill-posed) inverse retrieval algorithm. We lastly recover the high-resolution lifetime image ${\tau_{\text{HR}}}^{*}$ by minimizing the following cost function

$$\begin{array}{c} {\tau_{\text{HR}}}^{*}=\text{arg}{\text{min}}_{{\hat{\tau }}_{\text{HR}}}C\left({\hat{\tau }}_{\text{HR}}\right),\text{where}\\ C\left({\hat{\tau }}_{\text{HR}}\right)={|A{\hat{\tau }}_{\text{HR}}-\tau_{\text{LR}}|}_{2}^{2}+\gamma {|{\hat{\tau }}_{\text{HR}}-\tau_{\text{LP}}|}_{2}^{2}+\beta {|{\hat{\tau }}_{\text{HR}}-{\hat{\tau }}_{\text{GP}}|}_{2}^{2}+\alpha {|D{\hat{\tau }}_{\text{HR}}|}_{1}\\ \text{subject to}\;{\hat{\tau }}_{\text{HR}}\geq 0 \end{array}$$ (2)

The first term in $C\left({\hat{\tau }}_{\text{HR}}\right)$ ensures the data fidelity between the low-resolution measured lifetime image and the downsampled optimal high-resolution lifetime solution in each iteration. Prior constraints on the target high-resolution lifetime image are enforced through the second and the third data fidelity term, weighed by the factor γ and β, respectively, which are empirically optimized to yield best results. The fourth term is the *l*1 norm of the two-dimensional total variation (TV) evaluated on the high-resolution lifetime image and weighed by α (*59*). We consider the anisotropic form of the TV (*60*), and so the operator **D** represents the finite differences approximation of the horizontal and vertical image gradients. The complete workflow is visualized in movie S1.

### Dependence between lifetime and intensity

SiSIFUS priors exploit interdependence between fluorescence lifetime and intensity. Although these variables are interdependent at the single-molecule level via fluorescence quantum yield, this dependence is modulated by fluorophore concentration and other complex and often unpredictable biophysical mechanisms, thus necessitating statistical methods to create our priors.

Fluorescence quantum yield *Q* is the ratio of the number of emitted photons to the number absorbed. It depends on the radiative and nonradiative decay rates *k*_r_ and *k*_nr_ that depopulate excited molecules. The measured fluorescence lifetime τ also depends on these rates (*61*)

$$\begin{array}{c} Q=\frac{k_{r}}{k_{r}+k_{\text{nr}}}\\ \tau =\frac{1}{k_{r}+k_{\text{nr}}} \end{array}$$ (3)

therefore *Q* = *k*_r_τ for a single molecule. Across a given FOV, fluorescence intensity variations are given by the quantum yield of fluorophores (equivalently, fluorescence lifetime) multiplied by their absorbance (concentration × absorptivity × sample thickness). Absorbance is typically unknown and unpredictable; hence, it acts as a confounding variable in intensity-lifetime dependencies, so fluorescence intensity alone cannot give us full lifetime information. This means that two samples might have the same lifetime but completely different intensities, or vice versa.

However, across a single sample, fluorophore concentration typically varies slowly compared to lifetime and/or covaries with it on local scales, such that it is possible to build LPs that capture the resulting intensity-lifetime dependencies. Further, absorbance and lifetime often covary with cellular morphology, enabling us to create GPs. As a fail safe, if neither local nor global dependencies exist across a specific sample or subregion, TV minimization (a form of edge-preserving interpolation) in our inverse retrieval ensures that our method still performs at least as well as standard interpolation (see the Supplemental Materials for details).

#### Local prior

The LP relies on direct, pixelwise dependencies between lifetime and intensity on micrometer scales: Fig. 1 illustrates our workflow. If the images come from different detectors, then the lifetime and intensity images are first coregistered to match their fields of view. Figure 1A shows a sparse, low-resolution lifetime image (red-green-blue) overlayed on the corresponding intensity image (grayscale). The FOV is divided into windows, each containing a set of corresponding intensity-lifetime samples. These samples neighbor intensity pixels in the window center; hence, this window is used to create a prior for those pixels. In each window, the intensity and lifetime pairs are vectorized and fitted with a function, *f* (see Fig. 1B). Thus, our lifetime estimate $\hat{\tau }$ for pixel (λ*i* + *x*, λ*j* + *y*) is

$${\hat{\tau }}_{\lambda i+x,\lambda j+y}=f_{i,j}\left(I_{\lambda i+x,\lambda j+y}\right)$$ (4)

with samples *i* ∈ {0,1, …, *m* − 1} and *j* ∈ {0,1, …, *n* − 1} and *x* ≥ 0, *y* < λ. The functions *f*_*i*,*j*_ are fitted locally, not globally. Consequently, this procedure is repeated by sliding the window across the FOV, as shown in Fig. 1C.

#### Global prior

Images often contain multiple examples of similar features, with similar lifetime distributions, across the FOV. This motivates our development of GPs that exploit correlations between high-resolution morphology and lifetime.

Figure 2 shows our pipeline. We first extract intensity patches centered on our SPAD pixels, as shown in Fig. 2A. To deal with the relatively small number of patch-lifetime pairs contained in a single-sample image, we augment our training set. We use a commonly used dataset augmentation technique by reflecting and rotating the intensity windows in the training set. These operations increase our dataset eightfold, as shown in Fig. 2B. For high upsampling factors (8 × 8 and 16 × 16), we further augment the training set by estimating the lifetimes of the patches neighboring our sampled patches. We then label these patches with the same lifetime as their sampled neighbor. Our approach is visualized in Fig. 2B (see Materials and Methods for details).

Our GPs are designed to generalize new samples with previously unseen morphologies, morphology-lifetime dependencies, and lifetime ranges. A deep neural network (DNN), shown in Fig. 2B, is trained from scratch for each new sample on the intensity-patch inputs and lifetime labels obtained from the given microscope FOV. Consequently, different DNN initializations give slightly different predictions. To estimate high-resolution lifetime, we pass each intensity patch through our trained DNN, predicting the central lifetime value, as shown in Fig. 2C.

#### Quality metrics

We track reconstruction quality using three metrics: learned perceptual image patch similarity (LPIPS), structural similarity index measure (SSIM), and peak SNR (PSNR). LPIPS measures the distance between images in feature space. It has a minimum of 0 and grows with image dissimilarity (higher values are worse). SSIM tracks the similarity in luminance, contrast, and structure between two images; it is bound between −1 and 1, with larger values indicating better image similarity. Last, PSNR is a pixel-to-pixel comparison, where larger values are better. See the Supplementary Materials for details.

#### Sample 1: 16 × 16 (Madin-Darby canine kidney Flipper-TR)

We examined a validation sample of Madin-Darby canine kidney (MDCK) cells that had been treated with Flipper-TR dye (Spirochrome Inc.), which allows quantitation of tension in living, migrating cells. Data were acquired using a commercial LaVision BioTec TriM Scope system, using two-photon excitation scanning and detecting emission via a photomultiplier tube. The sample was imaged at 512 × 512 spatial points covering a 167-μm × 167-μm FOV and binned into 75 time bins, giving a 512 × 512 × 75 datacube. See Materials and Methods for details.

**Fig. 3.**
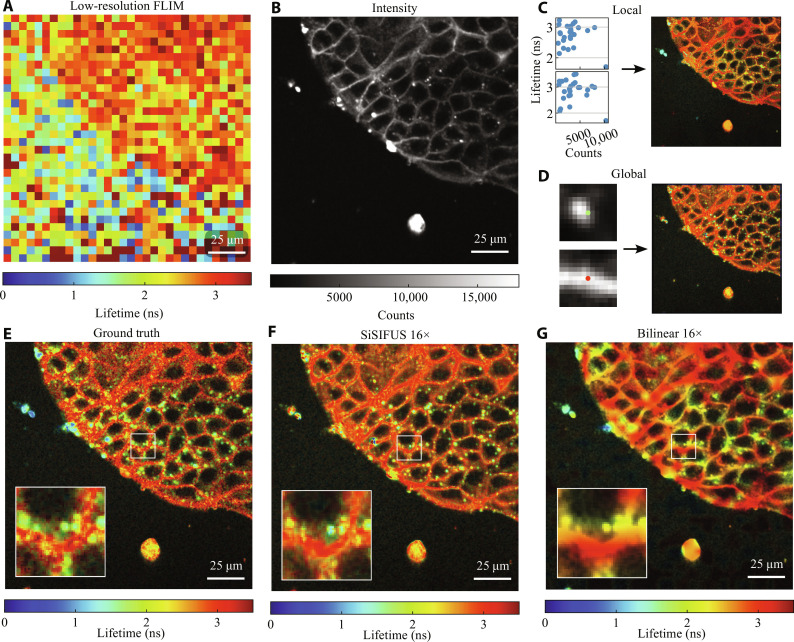
Upsampling (16 × 16) of MDCK cells. (**A**) Low-resolution fluorescence lifetime image (32 × 32) of MDCK cells expressing Flipper-TR dye. (**B**) Corresponding high-resolution intensity image (512 × 512) of the sample. (**C**) Windows (5 × 5) of low-resolution FLIM are fitted to corresponding intensity values to generate an LP image (two example windows are shown). (**D**) A GP image is generated from 13 × 13 intensity patches with central FLIM measurements (two examples are shown). (**E**) The GT high-resolution FLIM target, intensity-weighted for visualization. (**F**) The proposed method, upsampling the low-resolution measurement by a factor of 16 × 16. (**G**) Bilinear interpolation upsampling the FLIM measurement by 16 × 16.

Ground-truth (GT) fluorescence lifetime was estimated from this datacube using least-squares deconvolution. This was decimated 16-fold to give a low-resolution FLIM image, shown in Fig. 3A. The low-resolution FLIM image is severely undersampled; a lot of the detail was lost. Fluorescence intensity (Fig. 3B) was obtained in parallel, by summing the datacube along time. In the intensity image, we see that the probe mainly localized to two types of structures: small blobs (vesicles) and edges (cell membranes). In Fig. 3 (C and D), we show three examples of LP windows and corresponding GP patches, extracted as shown in Figs. 1B and 2A, respectively. Local (pixelwise) dependencies appear relatively weak; instead, the global (morphological) dependencies dominate, capturing the trend that vesicles have lower lifetimes than cell membranes across the FOV.

Figure 3E shows the GT lifetime, weighted with local contrast enhanced fluorescence intensity for visualization (details in the Supplementary Materials). In Fig. 3 (F and G), we show SiSIFUS and bilinear interpolation for the upsampling task. SiSIFUS automatically learns to distinguish between vesicles and cell membranes and labels them with different lifetimes, whereas interpolation fails to reconstruct fine details. SiSIFUS also maintains sharp boundaries between structures of different lifetimes, informed by the intensity image. SiSIFUS has an LPIPS of 0.24, an SSIM of 0.31, and a PSNR of 26 dB. Interpolation, instead, has an LPIPS of 0.48, an SSIM of 0.12, and a PSNR of 24 dB.

#### Sample 2: 8 × 8 (Convallaria acridine orange)

We applied SiSIFUS to a *Convallaria* rhizome sample dyed with acridine orange. The fluorescence lifetime datacube was recorded using our custom microscope setup, which uses a FLIMera 192 × 128–pixel SPAD array (HORIBA Scientific) (*62*) to image an 82-μm × 107-μm FOV, using 326 time bins. To compensate for the HORIBA camera’s asymmetric pixel layout, we scanned the sample twice with a pixel shift, giving a 192 × 256 × 326 datacube. Simultaneously, a high–spatial resolution scientific CMOS (sCMOS) camera registered a 2048-pixel × 2048-pixel image of the sample. The sCMOS camera is spatially coregistered to match the SPAD’s FOV and resolution (see Materials and Methods).

**Fig. 4.**
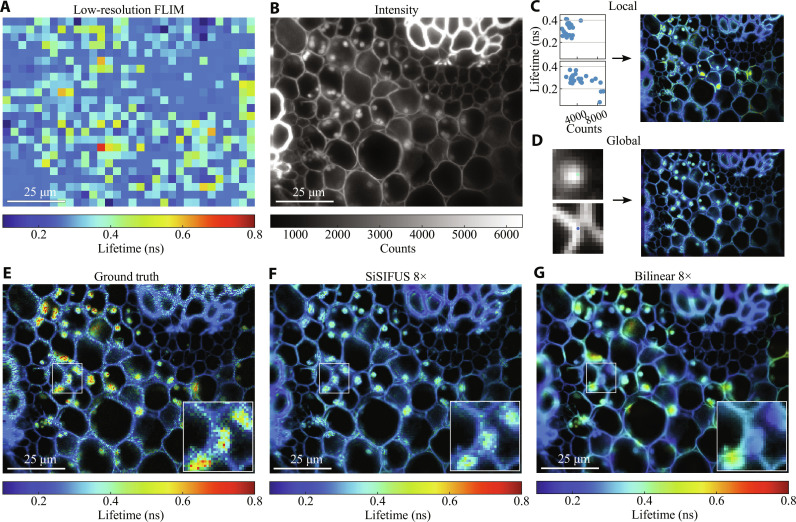
Upsampling (8 × 8) of *Convallaria* images. (**A**) Low-resolution fluorescence lifetime image (24 × 32) of a *Convallaria* rhizome sample stained with acridine orange, viewed under a wide-field microscope. (**B**) High-resolution intensity image (192 × 256). (**C**) Example 5 × 5 windows of low-resolution intensity versus FLIM, used for generating the LP shown on the right. (**D**) High-resolution intensity patches are labeled with lifetime, letting us create a GP. (**E** to **G**) GT, 8 × 8 SiSIFUS, and 8 × 8 bilinear interpolation of the data, weighted by local contrast enhanced intensity for visualization (see the Supplementary Materials for details).

The low-resolution FLIM and high-resolution intensity guide are shown in Fig. 4 (A and B). Figure 4 (C and D) shows example local and global dependencies and priors, respectively. Global dependencies appear to dominate local ones for this sample, with globules having shorter lifetimes than cell walls.

The 8 × 8 upsampling results in Fig. 4 (E to G) illustrate that SiSIFUS recognizes that globules tend to have higher lifetimes than cell walls, hence maintaining contrast between these structures more consistently than bilinear interpolation. We do note though that global SiSIFUS misses certain hotspots in the GT lifetime image (high lifetime, yellow/red-colored areas), likely because few globules in the training set have these lifetimes; hence, the model treats them as outliers. SiSIFUS achieves an LPIPS of 0.11, an SSIM of 0.21, and a PSNR of 16 dB. Bilinear interpolation has an LPIPS of 0.15, an SSIM of 0.29, and a PSNR of 16 dB.

#### Sample 3: 16 × 16 (SKOV3–Rac1-Raichu)

We further validate SiSIFUS on measurements of SKOV3 ovarian cancer cell samples expressing Rac-Raichu Clover-mCherry (see Materials and Methods). The GT images are acquired using our LaVision BioTec TriM Scope two-photon scanning system. The FOV was sampled on a 256 × 256 square grid covering 301-μm × 301-μm area. The temporal evolution was recorded using TCSPC with 75 time bins of 160-ps duration each, giving a fluorescence datacube of size 256 × 256 × 75.

**Fig. 5.**
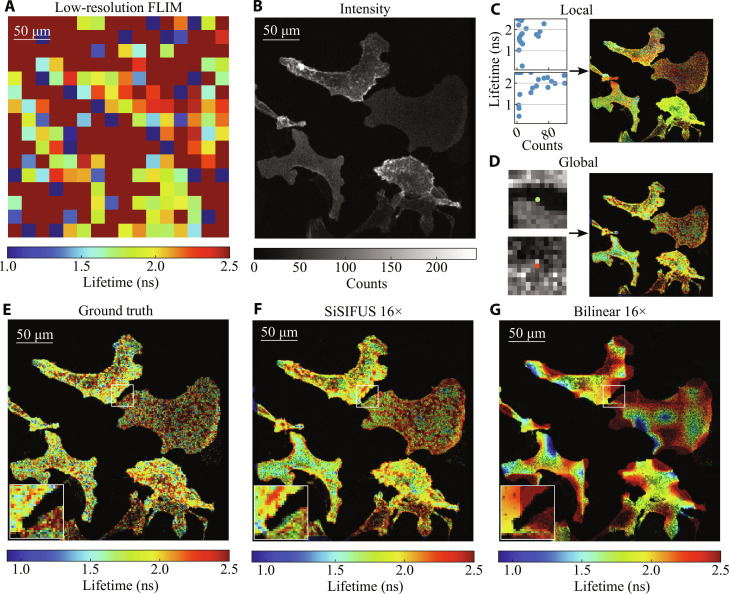
Upsampling (16 × 16) of SKOV3 cells. (**A**) Low-resolution fluorescence lifetime image (16 × 16) of an SKOV3 samples expressing Rac1-Raichu. (**B**) High-resolution intensity image (256 × 256). (**C** and **D**) Examples of local and global dependencies. (**E** to **G**) GT, 16 × 16 super-resolved, and 16 × 16 interpolated images.

The low-resolution fluorescence lifetime input is shown in Fig. 5A, alongside the high-resolution intensity guide in Fig. 5B. Figure 5 (C and D) shows a set of local windows and the LP, as well as global patches and the GP, respectively.

Local dependencies exhibit plateauing positive correlations and seem to flatten fluorescence lifetimes across the different cells, capturing intercellular dynamics. GPs instead capture intracellular dynamics, showing that fluorescence lifetime is mostly uniform within cells, with some patterned textures. Since the upsampling factor is high (16 × 16), our algorithm prioritizes GPs.

Figure 5 (E to G) shows the GT compared to 16 × 16 SiSIFUS and bilinear interpolation; SiSIFUS gives a better estimate. SiSIFUS reconstruction has an LPIPS to the GT of 0.15, an SSIM of 0.08, and a PSNR of 12 dB. In contrast, interpolation has an LPIPS of 0.34, an SSIM of 0.06, and a PSNR of 11 dB.

#### Sample 4: 8x8 (SKOV3 Rac1-Raichu)

We further validate SiSIFUS on wide-field images of SKOV3 ovarian cancer cells expressing Rac-Raichu Clover-mCherry, acquired with our custom FLIMera SPAD array and Zyla sCMOS setup (see Materials and Methods). The temporal evolution was recorded using TCSPC with 326 time bins, giving a fluorescence datacube of size 192 × 128 × 326. This image was decimated to give the low-resolution FLIM input.

**Fig. 6.**
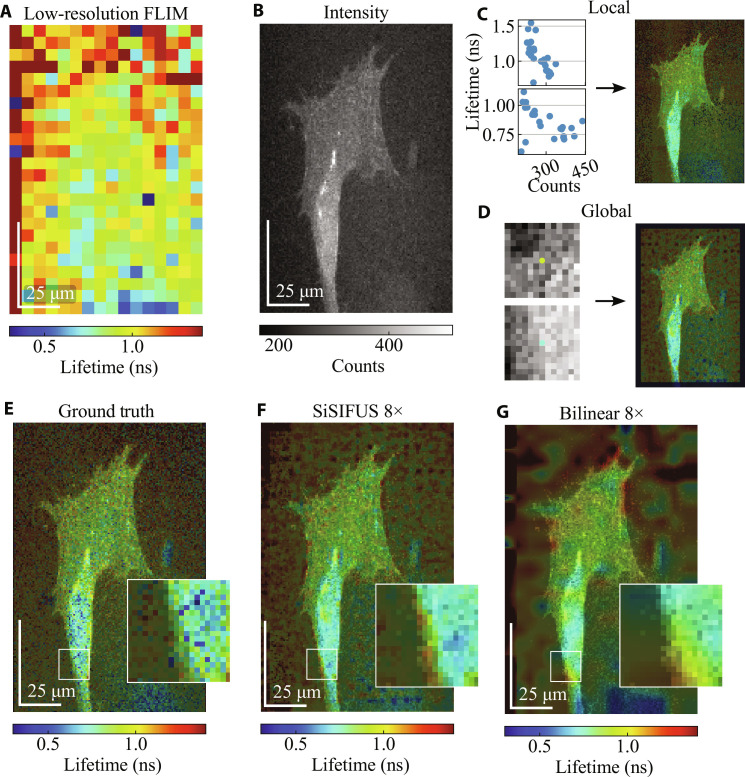
SR (8 × 8) of SKOV3 images. (**A**) Low-resolution FLIM image of an SKOV3 cell expressing the Rac1-Raichu probe (24 × 16). (**B**) The corresponding high-resolution fluorescence intensity image (192 × 128). (**C** and **D**) Comparison of local intensity– and global intensity–lifetime dependencies observed in this sample, and the corresponding local and GP images. (**E** to **G**) High-resolution GT FLIM, 8 × 8 SiSIFUS, and 8 × 8 bilinearly interpolated images, respectively.

Figure 6 shows our results. The low-resolution FLIM (Fig. 6A) and high-resolution intensity (Fig. 6B) are used to generate local and GPs, which are then fed into our inverse retrieval algorithm. Figure 6 (C and D) shows examples of local and global intensity lifetime dependencies and priors.

This sample shows nonlinear negative local interdependencies at most regions; SiSIFUS can exploit these to accurately determine the lifetime based on local intensity patterns. Conversely, global patch-lifetime dependencies are negligible. This is mainly because the FOV lacks repeating morphological features (in contrast to the MDCK and *Convallaria* samples in Figs. 3 and 4). Our algorithm prioritizes the LPs.

Last, Fig. 6 (E to G) shows the GT compared to SiSIFUS and bilinear interpolation. SiSIFUS succeeds in reconstructing the lifetime boundaries seen at the cell edges and also reconstructs the speckliness of the GT lifetime map, allowing the user to infer that there might be lifetime estimation uncertainty. Interpolation fails in these regards: Edges are blurred, and lifetime estimates appear smooth give the impression of structures that are absent in the GT. SiSIFUS yields an LPIPS of 0.31, an SSIM of 0.21, and a PSNR of 16 dB, whereas interpolation has an LPIPS of 0.56, an SSIM of 0.22, and a PSNR of 16 dB.

### Acquisition times

SiSIFUS provides an advantage in terms of acquisition times. For example, if we consider the case of measurements taken with our TriM Scope I (Figs. 3 and 5), the acquisition timescales linearly with pixel number as this is a galvo-scanning system. Therefore, we have an immediate advantage given by the SiSIFUS resolution enhancement factor that is applied. Specifically, in Fig. 3, where we apply 16 × 16 resolution enhancement, we have a 256× reduction in the number of points that need to be scanned and hence a 256× reduction in acquisition time. In a scanning system, we still, however, need to perform a second scan for the high-resolution intensity measurement, but this typically can be at substantially higher speed, of order 35× in our system (and this therefore remains the limiting factor). If we therefore consider the specific case of a 512 × 512 image (Fig. 3), the total acquisition time without SiSIFUS was 73 s and with SiSIFUS is 2.4 s allowing an acquisition rate of 0.4 frames s^−1^.

If, instead, we consider the case of measurements with an SPAD camera (in our case, the HORIBA FLIMera system; Figs. 4 and 6) this currently operates at 30 frames s^−1^, i.e., 33-ms acquisition time for an SiSIFUS image of any size (all pixels are acquired in parallel without any point-by-point scanning used in confocal imaging systems). We note that, in this case, the intensity CMOS image is acquired in parallel and hence does not add to the acquisition time.

We may compare this also with existing commercial systems, e.g., current B&H FLIM systems can measure 512 pixels × 512 pixels in 1 s (*63*) or previous work that operated directly with megapixel SPAD arrays in which, however, the smaller pixel size implied longer acquisition times to accumulate sufficient signal and was thus limited to ∼1-s acquisition times (*23*).

## DISCUSSION

We introduce SiSIFUS, a robust, data-fusion pipeline based on prior-augmented inverse retrieval for upsampling fluorescence lifetime images. We create two classes of priors that explicitly exploit a high-resolution intensity image to provide approximations for the nonsampled data points in a fluorescence lifetime image. The goal of SiSIFUS is to provide a “physics-inspired” approach to image resolution enhancement that performs better than standard bilinear or similar interpolation methods.

LPs capture pixel-wise correlations between fluorescence lifetime and intensity. For this, we find a direct mapping from intensity values to lifetime in small, local neighborhoods and use this mapping to predict the lifetime of intensity pixels that lack corresponding lifetime pixels. This allows SiSIFUS to maintain sharp spatial boundaries, tracking the boundaries of our intensity image. The LP is limited by measurement noise and sampling frequency. Since structures of similar intensity are assigned the same lifetime, undersampled regions may receive homogeneous lifetime estimates with sharp boundaries, as seen in the leftmost cell in Fig. 5C. Noisy regions can instead artificially track the intensity of an image’s noise, as in Fig. 6C. TV minimization and the GP help combat these issues.

GPs capture interdependence between FLIM and intensity on a morphological level. This is achieved by learning a mapping (DNN) from intensity patches to central-pixel lifetime samples and then using mapping to predict the lifetime of patches that have no central lifetime measurements. Thus, we capture nonlinear correlations between the brightness and shape of intensity features and lifetime. In microscopic samples, there often exist strong global trends between these variables, allowing the model to predict the lifetimes of patches with unsampled centers. However, the GP has limited ability to distinguish between similar morphologies with different lifetimes, typically assigning them with the average lifetime of these structures. This causes outliers such as the low-lifetime globules on the left of Fig. 3 or the high-lifetime vesicles in Fig. 4 to be ignored in favor of global patterns, although the LP will still retain these outliers. Consequently, the GP is most beneficial for samples that contain many examples of similar morphological structures, where these structures share similar lifetime properties. Since GPs capture sample-dependent properties, they can be further exploited when the same sample is imaged across multiple regions of interest (for instance, in a mosaic scan), by stacking intensity patches with corresponding lifetime labels into a common training set to improve generalization.

The results demonstrate the fact that the introduction of the priors in the TV-based inverse retrieval algorithm makes the latter a tractable problem. SiSIFUS gives the upsampled lifetime image sharp spatial features by extracting spatial information from an intensity image. This feature similarity is shown by perceptual metrics such as LPIPS, as features such as edges, speckly textures, and object shapes are captured by SiSIFUS but not by interpolation. In contrast, pixelwise metrics penalize pixel-to-pixel estimation noise heavily, making them more lenient toward blurred, interpolated images than SiSIFUS. We therefore prefer the LPIPS metric (designed to measure image quality in a similar way to human perception) and used it to optimize our hyperparameters in validation. We note that Figs. 4 to 6 were used as validation images that allowed us to optimize all hyperparameters [LP and GP window sizes, LP function, GP model architecture, epochs and learning rate, alternating direction method of multipliers (ADMM) iterations, and loss function weights). These were then fixed and used to generate the images in Fig. 3.

It is worth emphasizing that SiSIFUS is currently not implemented simultaneously with high-speed measurements such as the aforementioned SPAD video of 30 frames s^−1^, limiting its use for direct feedback or real-time diagnostics, such as intraoperative imaging. However, it enhances measurement speed by sampling fewer lifetime points and estimating lost information afterward. Equivalently, this mitigates phototoxicity and photodamage by illuminating the sample with less light, useful for imaging live samples in research or biopsy.

Despite these limitations, SiSIFUS offers notable potential for various applications beyond its current validation scope of FLIM. It could be applied to tasks involving disparate image types, but exhibiting local or global correlations underscore its versatility and applicability in diverse research settings.

A key feature that we believe will be beneficial in any such approach is that image reconstruction is never based on statistical inference from other images—only the single-image samples acquired from the two cameras are used thus strongly reducing or eliminating artifacts that may occur, for example, in other machine-learned approaches that do indeed rely on large sets of additional data and images and thus representing a potential point of failure that is of concern in many applications.

## MATERIALS AND METHODS

### Experimental design

#### Mammalian cell culture conditions

Both the SKOV3 ovarian cancer cells and the MDCK cells were maintained in Dulbecco’s modified Eagle’s medium (DMEM) supplemented with 10% fetal bovine serum (FBS), 2 mM l-glutamine, and 1× penicillin-streptomycin (PenStrep). Cell lines were maintained in 10-cm dishes at 37°C and 5% CO_2_.

SKOV3 cells were transfected in the morning using Amaxa Nucleofector (Lonza) kit V, program V-001 with either 5 μg of Raichu-Rac1 Clover-mCherry or pcDNA3.1-mClover DNA [adapted from (*64*)] following the manufacturer’s guidelines and replated on 6-cm tissue culture (TC)-treated dishes at 37°C and 5% CO_2_. For live-cell imaging, cells were collected and replated onto 35-mm glass bottom MatTek dishes that were previously coated overnight with laminin (10μg ml^−1^) diluted in phosphate-buffered saline (PBS). These were left overnight at 37°C and 5% CO_2_.

The next morning before imaging, the dishes were washed twice with prewarmed PBS and replaced with prewarmed FluoroBrite DMEM supplemented with 10% FBS, 2 mM l-glutamine and 1× PenStrep. For fixed-cell imaging, the cells were collected and replated onto 22-mm glass coverslips that were previously coated overnight with laminin (10μg ml^−1^) diluted in PBS. These were left overnight at 37°C and 5% CO_2_. The next day, these cells were fixed in 4% paraformaldehyde for 10 min and washed with PBS and mounted using Fluromount-G (Southern Biotech).

The MDCK cells were trypsinized and plated on 35-mm glass-bottom MatTek dishes and left to settle for 4 hours. Flipper-TR probe (Cytoskeleton, CY-SC020) was resuspended in 50 μl of anhydrous dimethyl sulfoxide as per the manufacturer’s instructions to yield a stock of 1 mM. Flipper-TR was diluted in culture medium to 2 μM and incubated on the cells overnight at 37°C and 5% CO_2_.

The next morning before imaging, the dishes were washed twice with prewarmed PBS and replaced with prewarmed FluoroBrite DMEM (Thermo Fisher Scientific, A1896701) supplemented with 10% FBS, 2 mM l-glutamine, 1× PenStrep, and 2 μM Flipper-TR.

#### Multiphoton raster-scanning time-domain FLIM: Experimental setup details

For the dataset shown in Fig. 5, cells were left to equilibrate on a heated microscope insert at 37°C and perfused with 5% CO_2_ before imaging. Images were acquired in the dark using a multiphoton LaVision TRIM scan head mounted on a Nikon Eclipse inverted microscope with a 20× water objective. Illumination is provided by a Ti:sapphire femtosecond laser (Coherent Chameleon Ultra II) used at 920 nm (12% power). The fluorescence signal was passed through band pass filters of 525/50-nm emission and acquired using a FLIM X-16 Bioimaging Detector TCSPC FLIM system (LaVision BioTec). A 301-μm × 301-μm FOV corresponding to 256 pixels × 256 pixels was imaged at 600 Hz with a 10-line average in a total acquisition time of 5199 ms.

For the dataset shown in Fig. 3, cells were left to equilibrate on a heated microscope insert at 37°C and perfused with 5% CO_2_ before imaging. Images were acquired in the dark using a multiphoton LaVision TRIM scan head mounted on a Nikon Eclipse inverted microscope with a Nikon Apo 60× oil objective, 1.4 numerical aperture. Illumination is provided by a Ti:sapphire femtosecond laser used at 970 nm (8% power) with an acquisition delay of 5.440 ns. The fluorescence signal was passed through emission band pass filters of 600/60 nm and acquired using a FLIM X-16 Bioimaging Detector TCSPC FLIM system (LaVision BioTec).

A 163-μm × 163-μm FOV correlating to 512 pixels × 512 pixels was imaged at 600 Hz with a 70-line average for a total acquisition time of 72,575 ms (high resolution). A total of 100 high- and low-resolution images taken from three independent experiments. Background images (high and low resolutions) were obtained by closing the scan head using the above settings. Instrument response function was obtained using carbon nanorods with the above settings and a 1% laser power.

#### Wide-field time-domain FLIM: Experimental setup details

For the datasets shown in Figs. 4 and 6, a custom microscope system was built using high–spatial resolution sCMOS sensor (Andor’s Zyla) and the FLIMera SPAD array sensor. Spatial registration was achieved by identifying a set of four coregistered points on the SPAD and CMOS and mapping the CMOS image with a perspective transformation to match the FOV of the SPAD image. See the Supplementary Materials for a schematic of the experimental setup.

### Statistical analysis

#### Inverse retrieval algorithm

The optimization is implemented using the ADMM algorithm. For this, the minimization in Eq. 2 can be reformulated as

$$\begin{array}{c} \tau_{{\text{HR}}^{*}}=\underset{{\hat{\tau }}_{\text{HR}}}{\text{argmin}}{\left\|A{\hat{\tau }}_{\text{HR}}-\tau_{\text{LR}}\right\|}_{2}^{2}+\gamma {\left\|{\hat{\tau }}_{\text{HR}}-{\hat{\tau }}_{\text{LP}}\right\|}_{2}^{2}+\\ \beta {\left\|{\hat{\tau }}_{\text{HR}}-{\hat{\tau }}_{\text{GP}}\right\|}_{2}^{2}+\alpha {||z||}_{1}\;\text{subject to}\;A{\hat{\tau }}_{\text{HR}}-z=0\;\text{and}\;{\hat{\tau }}_{\text{HR}}\geq 0 \end{array}$$ (5)

The augmented Lagrangian for this problem can be written as

$$\begin{array}{c} L_{\rho }\left({\hat{\tau }}_{\text{HR}},z,y\right)={\left\|A{\hat{\tau }}_{\text{HR}}-\tau_{\text{LR}}\right\|}_{2}^{2}+\gamma {\left\|{\hat{\tau }}_{\text{HR}}-{\hat{\tau }}_{\text{LP}}\right\|}_{2}^{2}+\beta {\left\|{\hat{\tau }}_{\text{HR}}-{\hat{\tau }}_{\text{GP}}\right\|}_{2}^{2}+\\ \alpha {||z||}_{1}+y\left(D{\hat{\tau }}_{\text{HR}}-z\right)+\rho /2{\left\|D{\hat{\tau }}_{\text{HR}}-z\right\|}_{2}^{2} \end{array}$$ (6)

Here, *y* is the Lagrangian multiplier (or the dual variable), and ρ is the penalty parameter. The ADMM approach involves jointly minimizing the Lagrangian over all the primal variables, followed by the updates over the dual variables. The primal updates for the variables ${\hat{\tau }}_{\text{HR}}$ and *z* are given by

$$\begin{array}{c} {{\hat{\tau }}_{\text{HR}}}_{k+1}\leftarrow \underset{{\hat{\tau }}_{\text{HR}}}{\text{argmin}}{\left\|A{\hat{\tau }}_{\text{HR}}-\tau_{\text{LR}}\right\|}_{2}^{2}+\gamma {\left\|{\hat{\tau }}_{\text{HR}}-{\hat{\tau }}_{\text{LP}}\right\|}_{2}^{2}+\\ \beta {\left\|{\hat{\tau }}_{\text{HR}}-{\hat{\tau }}_{\text{GP}}\right\|}_{2}^{2}+y_{k}\left(D{\hat{\tau }}_{\text{HR}}-z_{k}\right)+\rho /2{\left\|D{\hat{\tau }}_{\text{HR}}-z_{k}\right\|}_{2}^{2} \end{array}$$ (7)

$$z_{k+1}\leftarrow \underset{z}{\text{argmin}}\alpha {||z||}_{1}+y\left(D{\hat{\tau }}_{\text{HR}}-z\right)+\rho /2{\left\|D{{\hat{\tau }}_{\text{HR}}}_{k}-z\right\|}_{2}^{2}$$ (8)

The dual update is given by

$$y_{k+1}\leftarrow y_{k}+\rho \left(D{\hat{\tau }}_{\text{HR}}-z\right)$$ (9)

For the primal minimization updates in Eqs. 7 and 8, we use the standard optimization technique based on the fast iterative soft thresholding algorithm. Each iteration of the ADMM, hence, comprises 90 iterations of fast iterative soft thresholding algorithm for the ${\hat{\tau }}_{\text{HR}}$ variable update.

The weighting factor γ for the LP term in the cost function has been kept constant for all the cases, wherein γ = 0.1. The factor β on the other hand is varied for different upsampling factors such that it is 0.02 for 2× and 4× upsampling factors and 0.5 for higher upsampling factor of 8× and 16×. The GP cannot predict lifetimes within 6 pixels of the edges of the sample, since one cannot extract a 13 × 13 window centered on these pixels. Consequently, the GP’s contributions from these regions are removed from the IR reconstruction. A total of 20 iteration steps are used for minimizing the cost function; further iteration typically gives insignificant change in the solution. A Python implementation of the IR reconstruction for upsampling a 256 × 256 image to 512 × 512 (2× upsampling) takes ~80 s.

#### LP window size and function selection

In lieu of an analytical formula linking lifetime and intensity, the extent of the windows and the form of these local functions must be found empirically from the data. A set of window sizes, spanning from 2 × 2 to 8 × 8 were tested. Likewise, a set of different function forms were tried, including Gaussian processes with radial basis function (RBF) kernels, B-spline fitting, and interpolation (nearest, linear, and cubic). The best form was found by comparing mean upsampling metrics over a validation set of four biological samples (shown in Figs. 3 to 6) for four upsampling factors each (2×, 4×, 8×, and 16×) (see the Supplementary Materials for details). Windows (5 × 5) and linear interpolation yielded the best results; hence, these are used in all results shown in this work.

#### GP data augmentation and training

Our GPs are generated from a neural network trained on a training set of intensity patches. The training set includes all patches with a central lifetime estimate and their rotated and mirrored copies. Further, for high upsampling factors, their nearest neighbors are added to the training set (these neighbors are also in the test set), as well as the rotated and mirrored copies of these neighbors. Once trained, the network is used to evaluate all the original intensity patches, which includes all the training patches and all patches with unknown lifetimes. A 3 × 3 neighborhood gives 9× more windows to train on; combined with augmentation for rotation and reflection invariance, this yields 72× more data. This dataset augmentation method assumes that within the pixel pitch of the intensity image, lifetime varies slowly. This does not hold for every pixel; therefore, estimated labels have inherent uncertainty. Nonetheless, this form of blind labeling increases the diversity of the training input set. This was empirically found to be necessary for 8 × 8 and 16 × 16 upsampling not because of the upsampling factor but rather because the decimated low-resolution input was so small, that the training set size was a severe limitation.

The 13 × 13 training and testing patches are copied into two channels, producing 13 × 13 × 2 input instances for the neural network. One channel is normalized on a per-patch basis, drawing focus to the shape and texture of the patch’s content. The other channel is divided by the maximum of the original intensity image, maintaining absolute intensity variations.

For the results shown in this paper, the network was trained three times with different random initializations, and the prior of median quality was selected. We train on intensity patches 13 × 13 × 2 large, with ADAM (*65*), using a batch size of 100 over 150 epochs with mean absolute error as the training loss, on an NVIDIA GeForce RTX 2080 Ti. Training on a 125 × 125 FLIM image (i.e., 125 × 125 × 8 = 125,000 intensity patches due to 8× data augmentation for rotation and reflection invariance) takes ∼25 min of training on this hardware using TensorFlow, irrespective of the target image size. Trained DNNs tend to have negligible bias compared to the SD of prediction. Since they are trained from scratch on each new sample, their training and validation losses vary from sample to sample, upsampling factor to upsampling factor, and initialization to initialization. Example test set performances can be seen in inset of Figs. 3D, 4D, 5D, and 6D.

## Supplementary Materials

### This PDF file includes:

Supplementary Text Figs. S1 to S5 Legend for movie S1 References
