# Supplementary material for: Single-sample image-fusion upsampling of fluorescence lifetime images
Source: Sci Adv. 2024 May 23;10(21):eadn0139. doi: 10.1126/sciadv.adn0139 (PMC11114222; doi:10.1126/sciadv.adn0139)
Supplement: Supplementary file 1 — Supplementary Text Figs. S1 to S5 Legend for movie S1 References [file sciadv.adn0139_sm.pdf]

Science Advances
AAAS

Supplementary Materials for

Single-sample image-fusion upsampling of fluorescence lifetime images

Valentin Kapitany *et al.*

Corresponding author: Valentin Kapitany, valentin.kapitany@glasgow.ac.uk;
Daniele Faccio, daniele.faccio@glasgow.ac.uk

Sci. Adv. **10**, eadn0139 (2024)
DOI: 10.1126/sciadv.adn0139

The PDF file includes:

Supplementary Text
Figs. S1 to S5
Legend for movie S1
References

Other Supplementary Material for this manuscript includes the following:

Movie S1

1. FLUORESCENCE INTENSITY AND LIFETIME

Lifetime and quantum yield

Fluorescence lifetime is described in literature as being independent of fluorescent intensity (12, 66), and of fluorophore concentration (67) and excitation intensity. Here, we examine the context of these claims, and demonstrate the limitations of these generalisations.

In fluorescence, a photon excites a ground-state electron into an excited state, which then decays back to the ground state radiatively at a rate known as the decay rate. Other decay pathways compete with fluorescence, such as non-radiative decay and inter-system energy transfer between the fluorescent molecule and its environment. The probability of emitting a fluorescent photon per excitation event is called the quantum yield of fluorescence. Fluorescent intensity is the product of excitation intensity, the absorbance of the fluorophores (which depends strongly on their concentration) and fluorescence quantum yield.

The decay rate is the inverse of fluorescence lifetime, which is the expected time that an electron spends in the excited state before decaying via fluorescence. This is an intrinsic property of the molecule, and thus, is assumed to be independent of factors like fluorophore concentration. Consequently, fluorescence lifetime can be used to distinguish between different molecule populations.

However, fluorophores interact with their environment. The environment, in turn, can modulate both the excitation and emission pathways, changing both intensity and lifetime. Excitation can be enhanced or quenched by metallic surfaces or particles within the sample such as silver (68) via plasmonic resonance. Emission is modulated via nonradiative (or alternative) decay pathways, quenching the molecule's radiative fluorescence as well as its lifetime, as derived in the main section of the paper.

Fluorescence intensity

An imaging system generates a fluorescence intensity signal that depends on the spectral radiance $L_f(\lambda_0)$ of the sample and the net photon detection efficiency $PDE(\lambda_0)$ of the imaging system.

Let us consider a thin sample within the focal length of the optical system, using an epifluorescence setup. Using nomenclature from (69), the spectral radiance $L_f(\lambda_o)[Wsr^{-1}m^{-2}nm^{-1}]$ emitted by the sample at wavelength λ_o from excitation light at λ_x is given by:

$$L_f(\lambda_o) = I_x\tilde{N}\Omega\epsilon(\lambda_x)Q(\tau,\lambda_o,\lambda_x) \quad (1)$$

where I_x is the incident excitation power $[W]$, $\tilde{N}(x,y)$ is the 2D concentration of fluorophores $[m^2]$ (the integral of the 3D concentration N of fluorophores along the length of the sample along the

optical axis z $\widetilde{N}(x, y) = \int_z N(x, y, z)dz$, Ω is the solid angle through which emitted light is collected from the sample $[sr]$, $\epsilon(\lambda_x)$ is the absorptivity of the fluorophore $[m^2]$ as per the Beer-Lambert Law, $Q(\tau, \lambda_o, \lambda_x)$ is the lifetime-dependent, spectral quantum yield of fluorescence.

This spectral radiance is imaged onto a detector that has a response $R[AW^{-1}]$ using a system with some étendue $\Gamma[m^2 sr]$. To obtain the signal generated by the emission spectrum, we must integrate over the emission spectrum, giving:

$$s_o = \int_{\lambda_o} L_f(\lambda_o)\Gamma R(\lambda_o)d\lambda_o \quad (2)$$

Substituting Eq. 1 into Eq. 2, and integrating over the acquisition time t_a gives us the measurement $M[C]$:

$$M = \int_{\lambda_o} \int_{t=0}^{t_a} I_x \widetilde{N}\Omega\epsilon(\lambda_x)Q(\tau, \lambda_o, \lambda_x)\Gamma R(\lambda_o)d\lambda_o dt \quad (3)$$

A fixed excitation and detection system allows us to calibrate the intensity I_x, the collection solid angle Ω, the etendue Γ, the response $R(\lambda_o)$, and the acquisition time t_a. Therefore, variations of intensity across the field of view will depend on molecular concentration $\widetilde{N}$ and absorptivity $\epsilon(\lambda_x)$ (whose product is the absorbance of the fluorophores), as well as the spectral quantum yield of fluorescence Q, which depends on fluorescence lifetime.

Dependence of intensity on lifetime

Absorbance and fluorescence lifetime appear to be unrelated, hence absorbance (ergo, fluorophore concentration) is an unpredictable confounding variable in intensity-lifetime dependencies.

Consequently, a fluorescence intensity measurement alone cannot give us full lifetime information. We therefore must use statistical priors to extract intensity-lifetime dependencies in the presence of biological confounding variables. A local prior is developed to extract dependencies when lifetime varies more rapidly in space than these confounding variables, or when they correlate with lifetime on local scales (either positively or inversely).

Further, many biological samples absorb fluorophores into particular subcellular compartments such as the cell membrane (70), vesicles (71) or the nucleus (72). This results in lifetime patterns that often track cellular morphology. A global prior is developed to extract such dependencies. If absorbance were completely randomly distributed (which tends not to be the case in real samples), our method would not offer improvement over interpolation, instead our methods might overfit on noise patterns. To prevent this, our algorithm uses TV-filtering to prevent very unrealistically noisy lifetime estimates.

The question is whether recognisable intensity-lifetime dependencies actually exist in biological samples, or if absorbance renders them unusable. Below, we consider a series of case studies of

fluorophore-environment interactions reported in literature, focusing on how these interactions modulate intensity and lifetime.

Case studies

Okabe et. al. (6) used a complex fluorescent molecule made of a thermosensitive unit, a hydrophilic unit and a fluorescent unit to monitor temperature. In response to higher temperature, the molecule becomes hydrophobic, curling up and increasing both fluorescence quantum yield (thereby, intensity) and fluorescence lifetime. Fluorophore concentration still affects fluorescence intensity; however, locally (in regions of uniform concentration or at organelle edges), intensity and lifetime covary. Indeed, the authors use this probe to demonstrate temperature differences between the nucleus and cytoplasm of cells, which are visibly differentiable on both the lifetime and intensity maps.

Ogikubo et. al. (7) used cellular auto-fluorescence of NADH to monitor intracellular pH. Their results show evident covariance of fluorescent intensity with fluorescence lifetime within cells; even though intensity is not a marker of pH, both intensity and fluorescence lifetime depend on the location of NADH within the cell. The reason for this is not explicitly explored, but different works have shown that the ratio of bound to free NADH depends on the local metabolism of the cell, which influences both the fluorescence lifetime and concentration of NADH autofluorescence (11). Correlations are similarly visible between NADH fluorescence intensity and lifetime in works by Stringari et. al. (12), as both of these parameters are covariate with cellular redox ratio.

Van der Linden et. al. (73) use FLIM as a tool for a quantitative measurement of calcium levels, independent of hardware. However, for a given hardware, fluorescent intensity spikes clearly show calcium spikes, even if they do not give absolute calcium concentrations on their own. Indeed, the authors demonstrate that their FLIM probe works by showing Supplementary videos of fluorescent intensity and lifetime side-by-side, which both show synchronised flickering. Lifetime and and intensity are strongly temporally correlated and are also locally correlated: cellular organoids have quasi uniform intensity and lifetime, both of which experience sudden gradients at organoid boundaries.

Verboogen et. al. (74) demonstrate a FLIM-FRET probe for the imaging of SNARE trafficking in dendrites. For example, F¨orster resonance energy transfer (FRET) relies on this phenomenon. In FRET, the fluorophore, known as the donor, is linked to another fluorophore known as the acceptor, such that their relative conformation can change. The donor molecule is excited and its fluorescence measured. If the donor and acceptor are far, the donor will decay as if it were alone. If the donor and acceptor are in close vicinity, excited electrons can transfer energy from the donor onto the acceptor molecule, providing an alternative decay path for electrons, decreasing both fluorescence quantum yield (thus, intensity) and lifetime.

Gorpas et. al. (14) use skin autofluorescence to determine qualitative boundaries between cancerous and healthy skin tissue. They demonstrate that FLIM shows skin cancer; they do so by overlaying an augmented reality image of FLIM onto a visibly melanated patch of skin, whose colour correlates strongly with its lifetime.

2. SAMPLING CONSIDERATIONS

SiSIFUS relies on either local dependencies, where there are gradual changes in confounding variables such as fluorophore concentration, or global dependencies between structure and lifetime. Local dependencies involve scenarios like free fluorophores in the cytoplasm, which diffuse to achieve a locally uniform density, or beads coated in fluorescent dyes. Another example is continuous cell membranes treated with diffusive dyes like Flipper TR.

Global dependencies assume that objects with similar shapes in the FOV have similar lifetimes, as illustrated in Fig. 3 and Fig. 5. For instance, in a scenario with a mixture of small and large fluorescent beads coated with different fluorophores, by sparsely sampling their lifetimes, we can infer the lifetimes of other beads in the image. Employing machine learning algorithms, SiSIFUS automates and enhances this pattern-matching process, foregoing manual matching. Capturing these dependencies is crucial for SiSIFUS; thus, sampling density must be adequate to measure them. Whether sampling locally or across the FOV, denser sampling is required for small or rare biological or mechanical structures. In general, the sparser the structures in the FOV and the greater the variety of distinct lifetimes expected to be resolved, the denser the sampling required. In the bead example, if a medium-sized bead is present but never sampled for lifetime, accurate estimation requires external knowledge. We illustrate the effect of

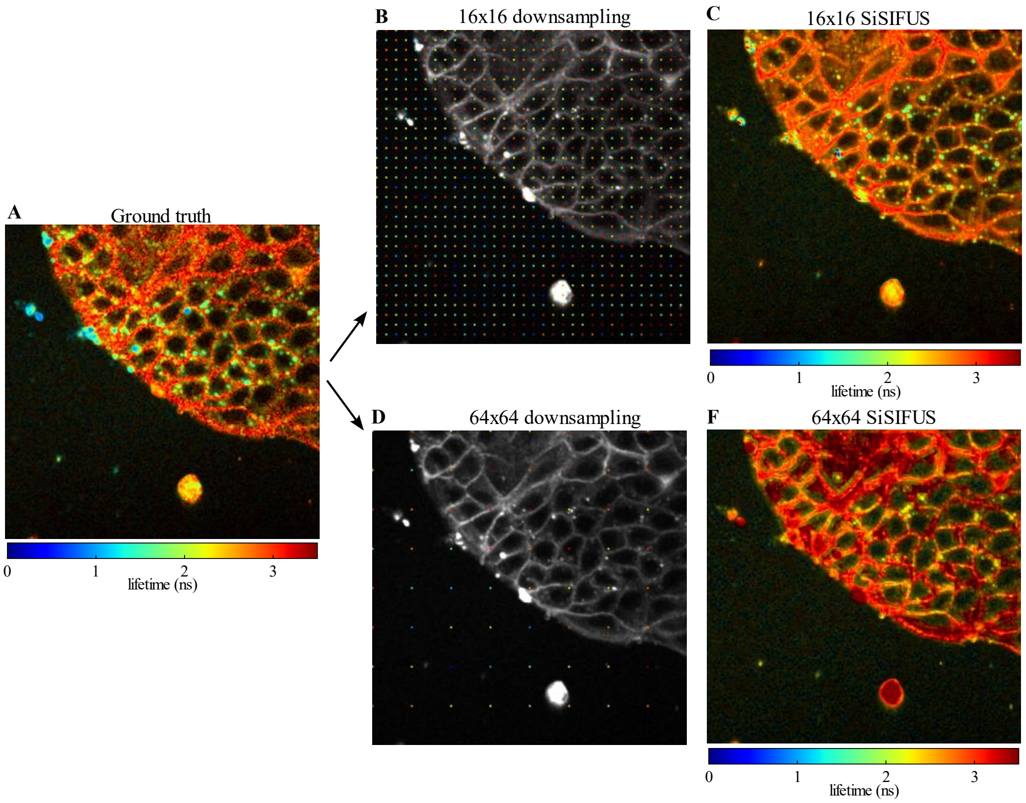

Figure S1. Illustration of the impact of under-sampling on image resolution. **(A)** The original image is 512x512 with a pixel pitch of 0.33µm. **(B)** Data is decimated by 16x16, with lifetime samples overlaid. **(C)** SiSIFUS can reasonably reconstruct the sample from intensity-lifetime pairs. **(D)** The ground truth lifetime is instead decimated by 64x64, resulting in only 8x8 lifetime measurements. **(E)** This extreme under-sampling causes SiSIFUS to fail in accurately recovering the lifetime distribution.

undersampling with a 64x64 super-resolved example of the MDCK-Flipper TR sample in Fig. 3 of the main text (from 8x8 to 512x512). Results are shown in Fig. S1.

3. LOCAL PRIOR

We performed a study to find the best window size and best function to map fluorescent intensity onto fluorescence lifetime with local priors. The window sizes were in the range 2 to 8, while the functions were a set of common schemes, ranging from B-splines (linear, quadratic and cubic), through regular interpolation (nearest, linear and cubic); and kriging (radial basis function Gaussian process fitting).

We applied these window sizes and functions to 4 samples (including the three shown in Figures 4-6) and 4 upsampling factors (2,4,8, and 16x). We evaluated the methods based on mean-absolute-error and LPIPS between the reconstruction and ground truth, averaged over these 4×4 scenarios. Our results are shown in Fig. S2 and Fig. S3. Based on these results, we decided to use a window size of 5 and linear interpolation for generating LPs.

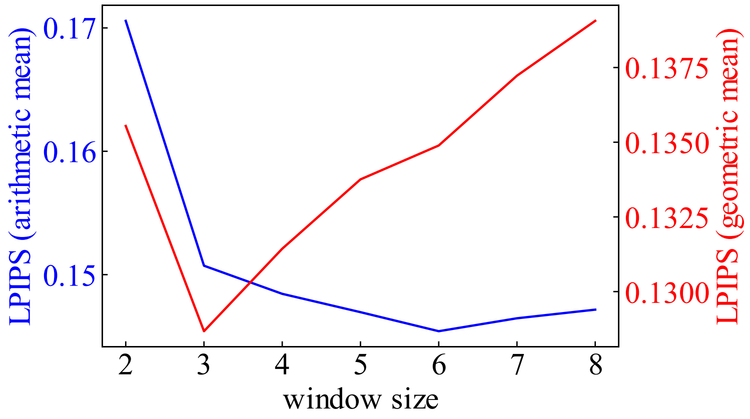

Figure S2. We found the mean LPIPS for priors generated using various window sizes, averaged across our 4 samples and 4 upsampling factors (2,4,8,16). We plot both the geometric and arithmetic mean.

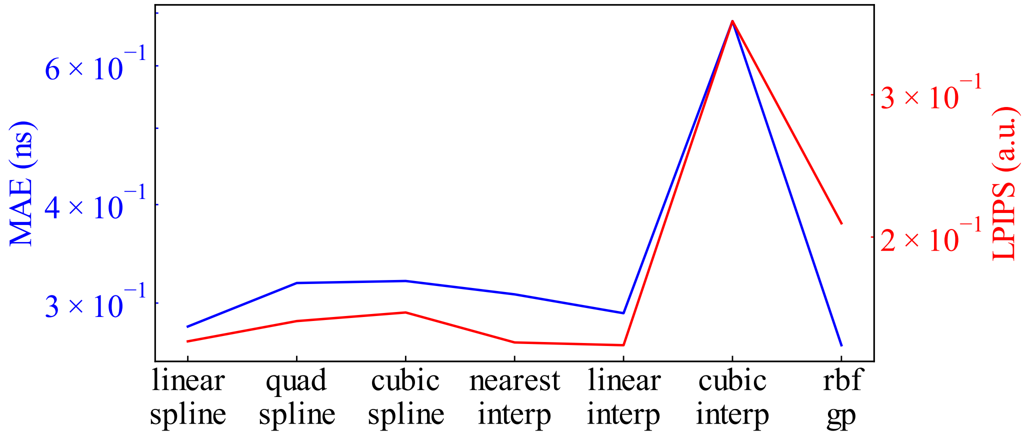

Figure S3: We found the mean MAE and LPIPS for priors generated using various LCP types, averaged across our 4 samples and 4 upsampling factors (2,4,8,16).

4. VISUALISATION

As stated in the main paper, we visualise our FLIM data by overlaying (weighting) it with local contrast enhanced intensity. This allows us to see lifetime patterns more clearly than using the raw lifetime image. For this, we first choose a colormap to plot the lifetime data in and use this colormap to convert it into an RGB image.

Separately, we apply Contrast Limited Adaptive Histogram Equalization (CLAHE) to the intensity image. This makes it to that the image has good contrast across the field of view, without saturating bright spots or rendering dark regions imperceptible. We then scale each channel of the RGB image with this contrast enhanced intensity, preserving the RGB image's color, but manipulating its brightness.

5. METRIC DETAILS

Pixelwise metrics such as mean absolute error (MAE), mean squared error (MSE) and peak signal-to-noise ratio (PSNR) are commonly used in image processing. Compared to MAE and MSE, PSNR is adjusted for image scale, letting it generalise image similarity across lifetime maps of different ranges. PSNR is guaranteed to favour the same method as MSE for a given sample, whilst allowing us to compare different samples as well. A quantitative and straight-forward pixel-to-pixel comparison, PSNR is valuable for automated tasks, however, a widely known issue of metrics is how poorly they reflect the human perspective of image similarity. A particularly infamous example is blurring, which produces relatively low pixelwise errors even when deteriorating image quality severely.

To address this, simple perceptual metrics such as the structural similarity index measure (SSIM) and multiscale SSIM (MS-SSIM) have been proposed. Indeed, SSIM is more sensitive to blurring than MSE and less sensitive to noise (75), akin to human perception. That said, SSIM and MS-SSIM are strongly correlated with MSE; in fact, their 'luminance' term is equivalent to MSE. Further, a statistical evaluation (76) found that, while SSIM correlates with human perceptual score to a factor of 0.9393, the PSNR (equivalently, MSE) baseline yields a score of 0.8709. This suggests that structural metrics, while an improvement on pixelwise metrics, still can be improved upon. In our study, we evaluate SSIM on windows of size 25.

One of the early realisations regarding convolutional neural networks was that the first few convolutional layers in a machine learning architecture tend to perform feature extraction, of growing abstractive complexity with increasing layer depth. This understanding led to the development of learned perceptual image patch similarity (LPIPS) by Zhang et. al. (77). The authors discovered that distance in the feature space of some image-trained neural network correlates well with human perception. Since then, LPIPS has gained widespread traction, for

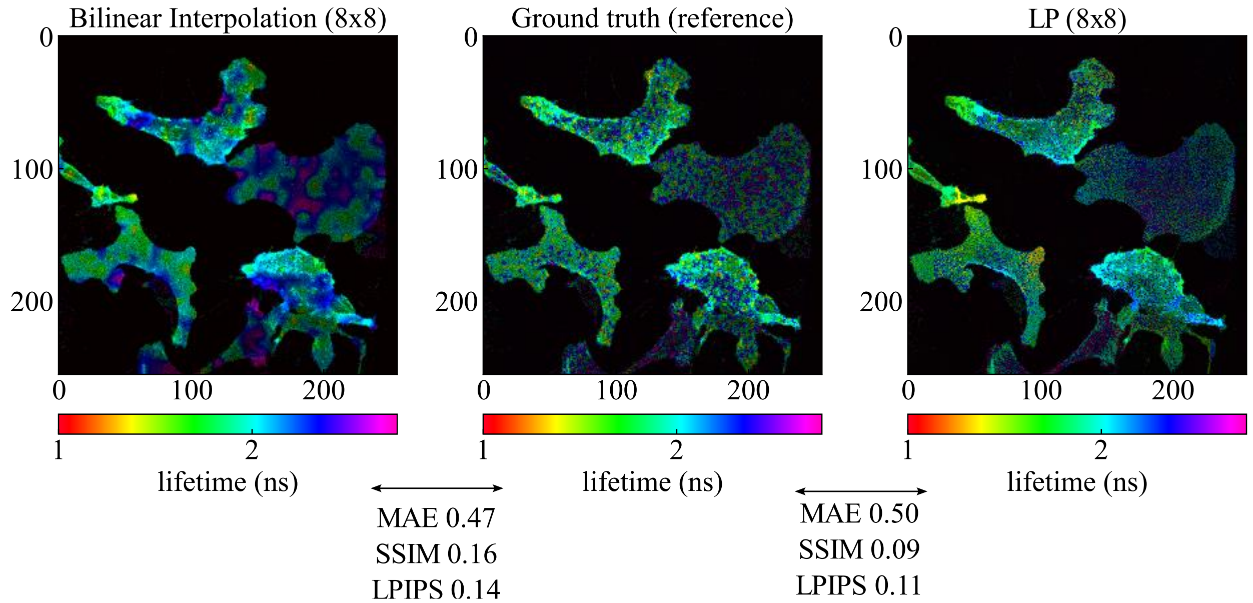

Figure S4. We show a comparison of interpolation vs an LP for 8 × 8 upsampling of a Rac-Raichu sample. Left to right: bilinear upsampling; the ground truth lifetime; LP. Both MAE and SSIM favour interpolation over the LP. However, the interpolated image is blurred and has artefacts matching the low-resolution sample grid, so, a user would likely say the LP is closer to the ground truth. LPIPS captures this perceptual similarity, favouring the LP.

uses LPIPS in training its autoencoder structure (78). The authors have added a Python implementation via the lpips package (79) including 3 pre-trained neural networks (In our works, instance the recently published image-to-image and text-to-image benchmark, Stable Diffusion, we use the AlexNet network). LPIPS is open-source and provides fixed-weight neural networks with a fixed protocol for calculating image similarity, which makes our LPIPS values exactly replicable.

We show an example from experimental data to illustrate the disparity between human vision and metrics with pixelwise components in Fig. S4.

6. EXPERIMENTAL SET-UP DETALS

The SPAD array datasets shown in Fig. 2 and Fig. 5 of the main paper were obtained by our bespoke microscope system comprising of the 192×128 pixels SPAD array sensor (FLIMera) and the sCMOS sensor (Andor Zyla), shown in Fig. S5. The system is a widefield epifluorescence setup, observing the sample using a 60x 1.4NA Nikon oil objective, alongside a 250mm focal length tube lens for the Zyla sensor and a 89.9mm focal length tube lens for the SPAD sensor. In this arrangement, we obtain 192 × 128 × 326 datacube for the dataset shown in Fig. 2. However, the detector’s pixel layout is such that two-columns of SPAD active areas are followed by a ‘dead space’ which is two pixel-pitches wide. For the dataset shown in Fig. 5 of the main paper, we correct for this irregular sampling. The full FOV is sampled in two shots, such that the image plane is translated by two pixel-pitches between the shots, and the outputs are then fused computationally. This yields a fluorescence datacube of size 192 × 256 × 326.

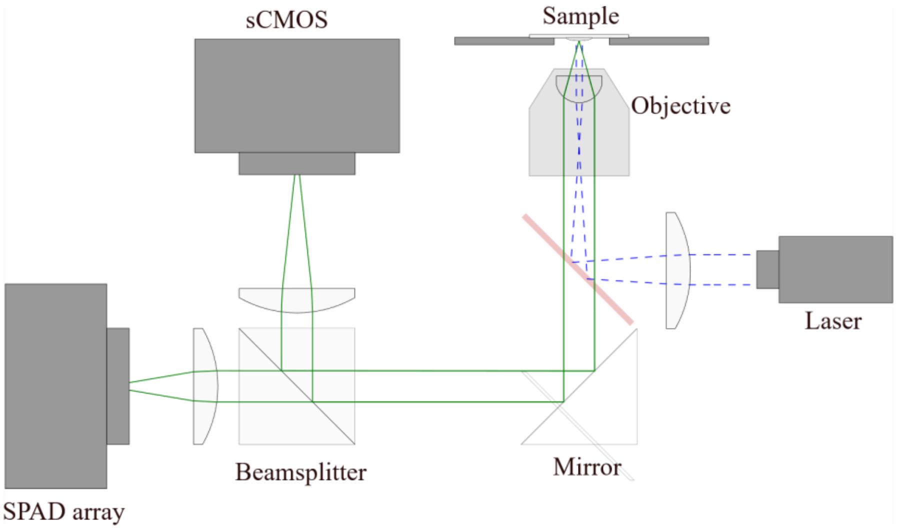

Figure S5. Schematic representation of the experimental set up: the sample is illuminated by a pulsed laser. The fluorescence signal from the sample is split by a dichroic beamsplitter, then collected and imaged on to the high spatial resolution sCMOS sensor and the low spatial resolution SPAD array sensor.

7. MOVIE S1

Visualisation of the complete SiSIFUS workflow, on a sample of Madin-Darby canine kidney cells treated with Flipper TR, imaged with the TriM Scope I system described in the main text. Lifetime is sampled 16x16 times sparser than intensity, with 32x32 pixel and 512x512 pixel resolutions, respectively. For upsampling, a local prior and global prior are independently generated from the low-res FLIM and high-res intensity image pair, then combined using our ADMM pipeline. Finally, the results are compared against the ground truth and interpolation, demonstrating how our method resolves finer spatial details than standard interpolation without external training data.

REFERENCES AND NOTES

1. I. Georgakoudi, K. P. Quinn, Optical imaging using endogenous contrast to assess metabolic state, *Annu. Rev. Biomed. Eng.* **14**, 351–367 (2012).
2. C. Stringari, H. Wang, M. Geyfman, V. Crosignani, V. Kumar, J. S. Takahashi, B. Andersen, E. Gratton, In vivo single-cell detection of metabolic oscillations in stem cells, *Cell Rep.* **10**, 1–7 (2015).
3. C. Stringari, L. Abdeladim, G. Malkinson, P. Mahou, X. Solinas, I. Lamarre, S. Brizion, J.-B. Galey, W. Supatto, R. Legouis, A.-M. Pena, E. Peaurepaire, Multicolor two-photon imaging of endogenous fluorophores in living tissues by wavelength mixing, *Sci. Rep.* **7**, 3792 (2017).
4. M. A. Yaseen, J. Sutin, W. Wu, B. Fu, H. Uhlirova, A. Devor, D. A. Boas, S. Sakadzic, Fluorescence lifetime microscopy of nadh distinguishes alterations in cerebral metabolism in vivo, *Biomed. Opt. Express* **8**, 2368–2385 (2017).
5. P. M. Schaefer, S. Kalinina, A. Rueck, C. A. von Arnim, B. von Einem, Nadh autofluorescence—A marker on its way to boost bioenergetic research, *Cytometry A* **95**, 34–46 (2019).
6. K. Okabe, N. Inada, C. Gota, Y. Harada, T. Funatsu, S. Uchiyama, Intracellular temperature mapping with a fluorescent polymeric thermometer and fluorescence lifetime imaging microscopy, *Nat. Commun.* **3**, 705 (2012).
7. S. Ogikubo, T. Nakabayashi, T. Adachi, M. S. Islam, T. Yoshizawa, M. Kinjo, N. Ohta, Intracellular pH sensing using autofluorescence lifetime microscopy, *J. Phys. Chem. B.* **115**, 10385–10390 (2011).
8. K. Suhling, Y. Teijeiro-Gonzalez, I. E. Steinmark, A. L. James, A. M. Economou, G. Yahioglu, A. Le Marois, L. M. Hirvonen, J. Nedbal, J. A. Levitt, P. H. Chung, C. A. Dreiss, A. J. Beavil, R. L. Beavil, E. Ortiz-Zapater, C. D. Lorenz, M. Parsons, A. Crnjar, B. Cornell, C. Molteni, Fluorescence lifetime imaging for viscosity and diffusion measurements, in *Multiphoton Microscopy in the Biomedical Sciences XIX* (SPIE, 2019), vol. 10882, pp. 115–123.
9. E. B. van Munster, T. W. J. Gadella, Fluorescence lifetime imaging microscopy (FLIM), in *Microscopy Techniques*, J. Rietdorf, Ed. (Springer, 2005), pp. 143–175.

10. B. T. Bajar, E. S. Wang, S. Zhang, M. Z. Lin, J. Chu, A guide to fluorescent protein fret pairs, *Sensors* **16**, 1488 (2016).
11. R. Datta, T. M. Heaster, J. T. Sharick, A. A. Gillette, M. C. Skala, Fluorescence lifetime imaging microscopy: Fundamentals and advances in instrumentation, analysis, and applications, *J. Biomed. Opt.* **25**, 071203 (2020).
12. M. Y. Berezin, S. Achilefu, Fluorescence lifetime measurements and biological imaging, *Chem. Rev.* **110**, 2641–2684 (2010).
13. Y. Sun, N. Hatami, M. Yee, J. Phipps, D. S. Elson, F. Gorin, R. J. Schrot, L. Marcu, Fluorescence lifetime imaging microscopy for brain tumor image-guided surgery, *J. Biomed. Opt.* **15**, 056022 (2010).
14. D. Gorpas, J. Phipps, J. Bec, D. Ma, S. Dochow, D. Yankelevich, J. Sorger, J. Popp, A. Bewley, and R. Gandour-Edwards, Autofluorescence lifetime augmented reality as a means for real-time robotic surgery guidance in human patients, *Sci. Rep.* **9**, 1187 (2019).
15. J. R. Lakowicz, Fluorescence-lifetime imaging microscopy, in *Principles of Fluorescence Spectroscopy*, J.R. Lakowicz, Ed. (Springer US, 2006), pp. 741–755.
16. J. R. Lakowicz, Time-domain lifetime measurements, in *Principles of Fluorescence Spectroscopy*, J. R. Lakowicz, Ed. (Springer US, 2006), pp. 130–131.
17. J. A. Jo, Q. Fang, L. Marcu, Ultrafast method for the analysis of fluorescence lifetime imaging microscopy data based on the laguerre expansion technique, *IEEE J. Sel. Top. Quantum Electron.* **11**, 835–845 (2005).
18. R. M. Ballew, J. N. Demas, An error analysis of the rapid lifetime determination method for the evaluation of single exponential decays, *Anal. Chem.* **61**, 30–33 (1989).
19. D. Elson, I. Munro, J. Requejo-Isidro, J. McGinty, C. Dunsby, N. Galletly, G. Stamp, M. Neil, M. Lever, P. Kellett, A. Dymoke-Bradshaw, J. Hares, P. M. W. French, Real-time time-domain fluorescence lifetime imaging including single-shot acquisition with a segmented optical image intensifier, *New J. Phys.* **6**, 180 (2004).
20. S. P. Poland, A. T. Erdogan, N. Krstajić, J. Levitt, V. Devauges, R. J. Walker, D. D.-U. Li, S. M. Ameer-Beg, R. K. Henderson, New high-speed centre of mass method

incorporating background subtraction for accurate determination of fluorescence lifetime, *Opt. Express* **24**, 6899–6915 (2016).

21. R. F. Laine, C. Poudel, C. F. Kaminski, A method for the fast and photon-efficient analysis of time-domain fluorescence lifetime image data over large dynamic ranges, *J. Microsc.* **287**, 138–147 (2022).
22. J. T. Smith, R. Yao, N. Sinsuebphon, A. Rudkouskaya, N. Un, J. Mazurkiewicz, M. Barroso, P. Yan, X. Intes, Fast fit-free analysis of fluorescence lifetime imaging via deep learning, *Proc. Natl. Acad. Sci. U S A* **116**, 24019–24030 (2019).
23. V. Zickus, M.-L. Wu, K. Morimoto, V. Kapitany, A. Fatima, A. Turpin, R. Insall, J. Whitelaw, L. Machesky, C. Bruschini, D. Faccio, E. Charbon, Fluorescence lifetime imaging with a megapixel spad camera and neural network lifetime estimation, *Sci. Rep.* **10**, 1 (2020).
24. V. Kapitany, V. Zickus, A. Fatima, G. Carles, D. Faccio, Single-shot time-folded fluorescence lifetime imaging, *Proc. Natl. Acad. Sci. U S A* **120**, e2214617120 (2023).
25. T. Knöpfel, C. Song, Optical voltage imaging in neurons: Moving from technology development to practical tool, *Nat. Rev. Neurosci.* **20**, 719–727 (2019).
26. L. Dvinskikh, H. Sparks, K. T. MacLeod, C. Dunsby, High-speed 2D light-sheet fluorescence microscopy enables quantification of spatially varying calcium dynamics in ventricular cardiomyocytes, *Front. Physiol.* **14**, 1079727 (2023).
27. J. McGinty, N. P. Galletly, C. Dunsby, I. Munro, D. S. Elson, J. Requejo-Isidro, P. Cohen, R. Ahmad, A. Forsyth, A. V. Thillainayagam, M. A. A. Neil, P. M. W. French, G. W. Stamp, Wide-field fluorescence lifetime imaging of cancer, *Biomed. Opt. Express* **1**, 627–640 (2010).
28. D. J. Kelly, S. C. Warren, S. Kumar, J. L. Lagarto, B. T. Dyer, A. Margineanu, E. W.-F. Lam, C. Dunsby, P. M. French, An automated multiwell plate reading flim microscope for live cell autofluorescence lifetime assays, *J. Innov. Opti. Health Sci.* **7**, 1450025 (2014).
29. A. J. Bowman, B. B. Klopfer, T. Juffmann, M. A. Kasevich, Electro-optic imaging enables efficient wide-field fluorescence lifetime microscopy, *Nat. Commun.* **10**, 4561 (2019).

30. A. J. Bowman, C. Huang, M. J. Schnitzer, M. A. Kasevich, Wide-field fluorescence lifetime imaging of neuron spiking and subthreshold activity in vivo, *Science* **380**, 1270–1275 (2023).
31. C. Bruschini, H. Homulle, I. M. Antolovic, S. Burri, E. Charbon, Single-photon avalanche diode imagers in biophotonics: Review and outlook, *Light: Sci. Appli.* **8**, 87 (2019).
32. Matplotlib, Interpolations for imshow.
33. S. Fadnavis, Image interpolation techniques in digital image processing: An overview, *Int. J. Eng. Res. Appl.* **4**, 70 (2014).
34. Q. Sun, J. Zhang, X. Dun, B. Ghanem, Y. Peng, W. Heidrich, End-to-end learned, optically coded super-resolution spad camera, *ACM Trans. Graph.* **39**, 1–14 (2020).
35. C. Callenberg, A. Lyons, D. den Brok, A. Fatima, A. Turpin, V. Zickus, L. Machesky, J. Whitelaw, D. Faccio, M. B. Hullin, Super-resolution time-resolved imaging using computational sensor fusion, *Sci. Rep.* **11**, 1689 (2021).
36. E. J. Candes, J. Romberg, T. Tao, Robust uncertainty principles: Exact signal reconstruction from highly incomplete frequency information, *IEEE Trans. Inform. Theor.* **52**, 489 (2006).
37. Q. Sun, X. Dun, Y. Peng, W. Heidrich, Depth and transient imaging with compressive spad array cameras, in *Proceedings of the IEEE Conference on Computer Vision and Pattern Recognition* (IEEE, 2018), pp. 273–282.
38. F. Soldevila, A. Lenz, A. Ghezzi, A. Farina, C. D'Andrea, E. Tajahuerce, Giga-voxel multidimensional fluorescence imaging combining single-pixel detection and data fusion, *Opt. Lett.* **46**, 4312 (2021), 4315.
39. N. Antipa, G. Kuo, R. Heckel, B. Mildenhall, E. Bostan, R. Ng, L. Waller, Diffusercam: Lensless single-exposure 3d imaging, *Optica* **5**, 1 (2018).
40. W. T. Freeman, T. R. Jones, E. C. Pasztor, Example-based super-resolution, *IEEE Comput. Graph. Appl.* **22**, 56–65 (2002).
41. H. Chang, D.-Y. Yeung, Y. Xiong, Super-resolution through neighbor embedding, in *Proceedings of the 2004 IEEE Computer Society Conference on Computer Vision and Pattern Recognition, 2004. CVPR 2004* (IEEE, 2004), vol. 1, pp. I–I.

42. J. Yang, J. Wright, T. S. Huang, Y. Ma, Image super-resolution via sparse representation, *IEEE Trans. Image Process.* **19**, 2861–2873 (2010).
43. R. Timofte, V. De Smet, L. Van Gool, Anchored neighborhood regression for fast example-based super-resolution, in *Proceedings of the IEEE International Conference on Computer Vision* (IEEE, 2013), pp. 1920–1927.
44. Z. Wang, J. Chen, S. C. Hoi, Deep learning for image super-resolution: A survey, *IEEE Trans. Pattern Anal. Mach. Intell.* **43**, 3365 (2020).
45. V. Kapitány, "AI for time-resolved imaging: From fluorescence lifetime to single-pixel time of flight," thesis, University of Glasgow (2023).
46. C. Dong, C. C. Loy, K. He, X. Tang, Learning a deep convolutional network for image super-resolution, in *Computer Vision–ECCV 2014* (Springer, 2014), pp. 184–199.
47. C. Dong, C. C. Loy, X. Tang, Accelerating the super-resolution convolutional neural network, in *Computer Vision– ECCV 2016* (Springer, 2016), pp. 391–407.
48. C. Ledig, L. Theis, F. Huszár, J. Caballero, A. Cunningham, A. Acosta, A. Aitken, A. Tejani, J. Totz, Z. Wang, W. Shi, Photo-realistic single image super-resolution using a generative adversarial network, in *Proceedings of the IEEE conference on computer vision and pattern recognition* (IEEE, 2017), pp. 4681–4690.
49. X. Wang, K. Yu, S. Wu, J. Gu, Y. Liu, C. Dong, Y. Qiao, C. Change Loy, ESRGAN: Enhanced super-resolution generative adversarial networks, in *Proceedings of the European Conference on Computer Vision (ECCV) Workshops* (Springer, 2018), pp. 63–79.
50. C. Saharia, J. Ho, W. Chan, T. Salimans, D. J. Fleet, M. Norouzi, Image super-resolution via iterative refinement, *IEEE Trans. Pattern Anal. Mach. Intell.* **45**, 1–14 (2022).
51. D. Glasner, S. Bagon, M. Irani, Super-resolution from a single image, in *2009 IEEE 12th International Conference on Computer Vision* (IEEE, 2009), pp. 349–356.
52. V. Mannam, Y. Zhang, X. Yuan, C. Ravasio, S. Howard, Machine learning for faster and smarter fluorescence lifetime imaging microscopy, *J. Phys.* **2**, 042005 (2020).
53. A. C. Quiros, N. Coudray, A. Yeaton, X. Yang, L. Chiriboga, A. Karimkhan, N. Narula, H. Pass, A. L. Moreira, J. L. Quesne, A. Tsirigos, K. Yuan, Self-supervised

learning unveils morphological clusters behind lung cancer types and prognosis. arXiv:2205.01931 (2022).

54. S.-C. Huang, A. Pareek, S. Seyyedi, I. Banerjee, and M. P. Lungren, Fusion of medical imaging and electronic health records using deep learning: A systematic review and implementation guidelines, *NPJ Dig. Med.* **3**, 136 (2020).
55. K.-H. Thung, P.-T. Yap, D. Shen, Multi-stage diagnosis of Alzheimer's disease with incomplete multimodal data via multi-task deep learning, in *International Workshop on Deep Learning in Medical Image Analysis* (Springer, 2017), pp. 160–168.
56. M. Person, M. Jensen, A. O. Smith, H. Gutierrez, Multimodal fusion object detection system for autonomous vehicles, *J. Dyn. Syst. Measur. Control* **141**, 071017 (2019).
57. T. Trzcinski, Multimodal social media video classification with deep neural networks, in *Photonics Applications in Astronomy, Communications, Industry, and High-Energy Physics Experiments 2018,* (SPIE, 2018), vol. 10808, pp. 879–886.
58. K. Samimi, D. E. Desa, W. Lin, K. Weiss, J. Li, J. Huisken, V. Miskolci, A. Huttenlocher, J. V. Chacko, A. Velten, J. D. Rogers, K. W. Eliceiri, M. C. Skala, Light-sheet autofluorescence lifetime imaging with a single-photon avalanche diode array, *J. Biomed. Opt.* **28**, 066502 (2023).
59. C. Ji, X. Wang, K. He, Y. Xue, Y. Li, L. Xin, W. Zhao, J. Tian, L. Sheng, Compressed fluorescence lifetime imaging via combined TV-based and deep priors, *PLOS ONE* **17**, e0271441 (2022).
60. A. Chambolle, An algorithm for total variation minimization and applications, *J. Math. Imag. Vision* **20**, 89–97 (2004).
61. J. R. Lakowicz, Introduction to Fluorescence, *Principles of Fluorescence Spectroscopy* (Springer, 2006) p 9.
62. R. K. Henderson, N. Johnston, F. M. D. Rocca, H. Chen, D. Day-Uei Li, G. Hungerford, R. Hirsch, D. Mcloskey, P. Yip, D. J. S. Birch, A 192 × 128 time correlated spad image sensor in 40-nm cmos technology, *IEEE J. Solid-State Circuits* **54**, 1907 (2019).
63. W. Becker, "SPC-QC-104: Precision FLIM and fast FLIM in one," Becker & Hickl GmbH, 2023; www.becker-hickl.com/literature/application-notes/spc-qc-104-precision-flim-and-fast-flim-in-one/ [accessed 16 January 2024].

64. R. E. Itoh, K. Kurokawa, Y. Ohba, H. Yoshizaki, N. Mochizuki, M. Matsuda, Activation of rac and cdc42 video imaged by fluorescent resonance energy transfer-based single-molecule probes in the membrane of living cells, *Mol. Cell. Biol.* **22**, 6582–6591 (2002).
65. D. P. Kingma, J. Ba, Adam: A method for stochastic optimization. arXiv:1412.6980 [cs.LG] (22 December 2014).
66. H. Szmacinski, J. R. Lakowicz, Fluorescence lifetime-based sensing and imaging, *Sens. Actuators B* **29**, 16–24 (1995).
67. K. Suhling, L. M. Hirvonen, J. A. Levitt, P.-H. Chung, C. Tregidgo, A. Le Marois, D. A. Rusakov, K. Zheng, S. Ameer-Beg, S. Poland, S. Coelho, R. Henderson, N. Krstajic, Fluorescence lifetime imaging (flim): Basic concepts and some recent developments, *Med. Photonics* **27**, 3–40 (2015).
68. J. R. Lakowicz, Radiative decay engineering: Biophysical and biomedical applications, *Anal. Biochem.* **298**, 1–24 (2001).
69. A. Schwartz, L. Wang, E. Early, A. Gaigalas, Y.-Z. Zhang, G. E. Marti, R. F. Vogt, Quantitating fluorescence intensity from fluorophore: The definition of mesf assignment, *J. Res. Natl. Inst. Stand. Technol.* **107**, 83–91 (2002).
70. M. Stockl, A. Herrmann, Detection of lipid domains in model and cell membranes by fluorescence lifetime imaging microscopy, *Biochim. et Biophys. Acta (BBA)-Biomembr.* **1798**, 1444–1456 (2010).
71. A. Pierzynska-Mach, P. A. Janowski, J. W. Dobrucki, Evaluation of acridine orange, lysotracker red, and quinacrine as fluorescent probes for long-term tracking of acidic vesicles, *Cytometry A* **85**, 729–737 (2014).
72. A. K. Estandarte, S. Botchway, C. Lynch, M. Yusuf, I. Robinson, The use of dapi fluorescence lifetime imaging for investigating chromatin condensation in human chromosomes, *Sci. Rep.* **6**, 1 (2016).
73. F. H. van der Linden, E. K. Mahlandt, J. J. Arts, J. Beumer, J. Puschhof, S. de Man, A. O. Chertkova, B. Ponsioen, H. Clevers, J. D. van Buul, M. Postma, T. W. J. Gadella Jr., J. Goedhart, A turquoise fluorescence lifetime-based biosensor for quantitative imaging of intracellular calcium, *Nat. Commun.* **12**, 1 (2021).

74. D. R. J. Verboogen, N. G. Mancha, M. Ter Beest, G. van den Bogaart, Fluorescence lifetime imaging microscopy reveals rerouting of snare trafficking driving dendritic cell activation, *eLife* **6**, e23525 (2017).
75. R. Dosselmann and X. D. Yang, A comprehensive assessment of the structural similarity index, *Signal, Imag. Video Process.* **5**, 81–91 (2011).
76. H. R. Sheikh, M. F. Sabir, and A. C. Bovik, A statistical evaluation of recent full reference image quality assessment algorithms, *IEEE Trans. Image Process.* **15**, 3440–3451 (2006).
77. R. Zhang, P. Isola, A. A. Efros, E. Shechtman, O. Wang, The unreasonable effectiveness of deep features as a perceptual metric, in *Proceedings of the IEEE Conference on Computer Vision and Pattern Recognition* (IEEE, 2018), pp. 586–595.
78. R. Rombach, A. Blattmann, D. Lorenz, P. Esser, B. Ommer, High-resolution image synthesis with latent diffusion models, in *Proceedings of the IEEE/CVF Conference on Computer Vision and Pattern Recognition* (IEEE, 2022), pp. 10684–10695.
79. R. Zhang, lpips 0.1.4 (2021). https://github.com/richzhang/PerceptualSimilarity/releases/tag/v0.1.4.
